# Development of breath test for pneumoconiosis: a case-control study

**DOI:** 10.1186/s12931-017-0661-3

**Published:** 2017-10-17

**Authors:** Hsiao-Yu Yang, Ruei-Hao Shie, Che-Jui Chang, Pau-Chung Chen

**Affiliations:** 1 Institute of Occupational Medicine and Industrial Hygiene, National Taiwan University College of Public Health, No. 17 Xuzhou Road, Taipei, 10055 Taiwan; 2 Department of Public Health, National Taiwan University College of Public Health, Taipei, Taiwan; 3 Department of Environmental and Occupational Medicine, National Taiwan University Hospital, Taipei, Taiwan; 4 Green Energy & Environmental Research Laboratories, Industrial Technology Research Institute, Hsinchu, Taiwan; 5 Department of Environmental and Occupational Medicine, National Taiwan University College of Medicine, Taipei, Taiwan

**Keywords:** Breath test, Volatile organic compounds, Lipid peroxidation, Pneumoconiosis, Breathomics

## Abstract

**Background:**

Lipid peroxidation plays an important role in the pathogenesis of pneumoconiosis. Volatile organic compounds (VOCs) generated from lipid peroxidation might be used to detect pneumoconiosis. The objective of this study was to develop a breath test for pneumoconiosis.

**Methods:**

A case-control study was designed. Breath and ambient air were analysed by gas chromatography/mass spectrometry. After blank correction to prevent contamination from ambient air, we used canonical discriminant analysis (CDA) to assess the discrimination accuracy and principal component analysis (PCA) to generate a prediction score. The prediction accuracy was calculated and validated using the International Classification of Radiographs of the Pneumoconiosis criteria combined with an abnormal pulmonary function test as a reference standard. We generated a receiver operator characteristic (ROC) curve and calculated the area under the ROC curve (AUC) to estimate the screening accuracy of the breath test.

**Results:**

We enrolled 200 stone workers. After excluding 5 subjects with asthma and 16 subjects who took steroids or nonsteroidal anti-inflammatory drugs, a total of 179 subjects were used in the final analyses, which included 25 cases and 154 controls. By CDA, 88.8% of subjects were correctly discriminated by their exposure status and the presence of pneumoconiosis. After excluding the VOCs of automobile exhaust and cigarette smoking, pentane and C5-C7 methylated alkanes constituted the major VOCs in the breath of persons with pneumoconiosis. Using the prediction score generated from PCA, the ROC-AUC was 0.88 (95% CI = 0.80—0.95), and the mean ROC-AUC of 5-fold cross-validation was 0.90. The breath test had good accuracy for pneumoconiosis diagnosis.

**Conclusion:**

The analysis of breath VOCs has potential in the screening of pneumoconiosis for its non-invasiveness and high accuracy. We suggest that a multi-centre study is warranted and that all procedures must be standardized before clinical application.

## Background

Pneumoconiosis is an important occupational lung disease that primarily consists of silicosis and asbestosis. Silicosis results from inhaling silica, which is a common component of rock and sand. Stone carvers, ceramic workers, quarry workers, miners, tunnel drillers, sand blasting workers, sand casting workers, and construction or demolition workers are at high risk for inhaling silica at work [1]. Silicosis in miners has shown a resurgence in recent years [2]. Asbestosis results from inhaling asbestos fibres that cause diffuse interstitial pulmonary fibrosis. The high-risk population includes shipyard workers, construction workers, asbestos textile workers, manufacturing brake lining workers, and asbestos miners or millers [1]. Although asbestos has been gradually banned in many countries, mortality due to asbestos exposure continues to increase in many developed countries. However, the diagnosis of early-stage pneumoconiosis is difficult in clinical practice [3]. The development of new diagnostic methods for pneumoconiosis is warranted.

Lipid peroxidation plays an important role in the pathological mechanisms of pneumoconiosis. When dust particles are inhaled and reach the alveolar space, the particles are phagocytosed by alveolar macrophages, where they induce the production of reactive oxygen species (ROS) and associated lipid peroxidation [4, 5]. Animal studies found that some volatile organic compounds (VOCs), such as pentane, are associated with lipid peroxidation [6]. We hypothesized that VOCs generated from lipid peroxidation could be used to detect pneumoconiosis. The objective of the study was to develop a breath test for pneumoconiosis by analysing exhaled VOCs.

## Methods

### Study subjects

We conducted a case-control study and recruited participants from stone workers in Hualien between March 2013 and July 2014. These stone workers processed jade artefacts, building materials, decorations, sculptures, vases, or urns and were exposed to a mixed dust environment. Our previous study found that these stone workers had an increased risk of pneumoconiosis [7].

### Occupational and medical histories

We obtained information on occupational history, socio-demographic indicators, smoking habits, and clinical history through a face-to-face interview using a validated questionnaire. The questionnaire was developed based on filed surveys that were conducted by occupational physicians and industrial hygienists, and it was pre-tested using senior stone workers in Hualien to correct any ambiguous wording. Cigarette smoking history was obtained, including the age at which smoking began, the age at which smoking ceased, and the average number of cigarettes smoked per day. A physician used a semi-structured questionnaire to review the subject’s medical history. The ATS-DLD-78-A questionnaire was used to obtain information on the subject’s symptoms of lung disease and their personal and family history of lung disease [8]. Disease was diagnosed by medical doctors and confirmed using the subject’s hospital and national health insurance records to rule out interstitial lung diseases caused by drugs, scleroderma, systemic lupus erythaematosus, rheumatoid arthritis, or any other autoimmune diseases [9, 10].

### Medical examinations

We arranged health examinations for all study subjects in a medical centre of Hualien. The medical examinations included physical examination, chest x-ray, pulmonary function test (PFT), fractional exhaled nitric oxide (FeNO) test, complete blood count (CBC), blood urine nitrogen (BUN), creatinine, fasting sugar, aspartate aminotransferase (AST), alanine aminotransferase (ALT), and urinary analyses. Chest X-rays were read by two physicians who were blind (masked) to the results of the other tests and clinical information. The profusion of pulmonary fibrosis in chest X-rays was graded in accordance with the International Labour Office (ILO)/International Classification of Radiographs of the Pneumoconioses (ICRP). The ILO Standard Digital Images (ILO 2011-D) were used to determine the profusion of pulmonary fibrosis, and a profusion score greater than or equal to 1/0 was defined as parenchymal abnormalities consistent with pneumoconiosis. We performed a standard PFT to measure forced vital capacity (FVC), forced expiratory volume in 1 second (FEV1), and forced expiratory flow between 25 and 75% of FVC (FEF25–75). An abnormal pulmonary function test was defined as FVC less than 80% of predicted value or FEV1/FVC less than 70%. PFT was performed by well-trained technicians. The measurement of FeNO followed the ARS/ERS recommendation [11]. Blood and urine samples were collected after overnight fasting.

### Case ascertainment

This study applied the ILO/ICRP criteria to diagnose pneumoconiosis. A case of pneumoconiosis was determined as having parenchymal abnormalities consistent with pneumoconiosis combined with abnormal pulmonary function test.

### Exclusion criteria

Diabetes mellitus [12], uraemia [13], asthma [14], chronic obstructive pulmonary disease (COPD) [15], pulmonary tuberculosis [16], cancers [17], infectious diseases [18], and the use of anti-inflammatory drugs might influence VOCs. We excluded subjects from the final analysis if they had an acute infection (defined as a white blood cell count greater than 10.0 × 10^3^/μL), poorly controlled diabetes mellitus (defined as a medical history of diabetes combined with a fasting blood glucose level greater than 250 mg/dL), uraemia (defined as an estimated glomerular filtration rate of less than 60 mL/min with uremic symptoms or a medical history of dialysis), COPD diagnosed by a physician, asthma (a medical history of asthma with a mean FeNO value greater than 50 ppb based on three repeated measurements, indicating significant airway inflammation [11] on the examination day), or were using steroids or nonsteroidal anti-inflammatory drugs (NSAIDs) on the examination day.

## Collection and analysis of breath air

This study applied single-breath sampling with a single expiratory capacity manoeuvre [19]. We used 1-l Entech Bottle-Vac canisters (Entech Instruments Inc., Simi Valley, CA, USA) to sample breath and background ambient air. Standardized preparation, sampling, and analytical procedures for the breath test included the following steps. (A) Step 1: before sampling, all canisters were cleaned in a qualified laboratory to prevent contamination. (B) Step 2: initial vacuum pressure was measured using a Micro-QT Valve Vacuum Check Gauge; a negative pressure of −30 Hg is required. (C) Step 3: collect 1 litre of background ambient air. (D) Step 4: collect 1 litre of breath sample. (E) Step 5: use the robotic headspace autosampler to introduce the samples into the column. (F) Step 6: pre-concentration. Finally, (G) Step 7: perform gas chromatograph-mass spectrometer (GC-MS) analysis. Because some VOCs might be generated from food or bacteria in the oral cavity [20], study subjects refrained from eating for 8 h and rinsed their mouths with distilled water prior to breath air sampling. All subjects sat in the room for at least 1 h before the breath samples were collected. The subjects exhaled 1 litre of breath into a disposable mouthpiece connected to a negative pressure canister. One litre of background ambient air was also sampled simultaneously. All canisters were immediately sent to an accredited laboratory for analysis. Gas samples were analysed within 48 h. Samples were analysed using an Entech 7500A Robotic Headspace Autosampler attached to an Entech 7150 Air/Headspace Preconcentrator using active solid-phase microextraction (SPME) (Entech Instruments Inc., Simi Valley, CA, USA) coupled to an Agilent 6890 N GC /5975C MS (Agilent Technologies, Santa Clara, CA, USA). VOCs were analysed qualitatively and quantitatively in accordance with U.S. Environmental Protection Agency Method TO-15 for the analysis of VOCs in air [21]. An internal standard spiking mixture containing bromochloromethane, chlorobenzene-d_5_, 1,4-difluorobenzene, and bromofluorobenzene at 10 ppmv each in humidified zero air was added to the sample for correction and calibration. The addition of 500 μL of this mixture to 500 mL of the sample resulted in a concentration of 10 ppbv. The internal standard was introduced into the focus trap during the collection time for all calibration, blank, and sample analyses. The volume of internal standard spiking mixture added to each analysis was the same for each run [21]. Internal standard responses and retention times were evaluated immediately after data acquisition. If the retention time for any internal standard changed by more than 20 s from the mean retention time over the initial calibration range or if the area response for any internal standard changed by more than 40% between the sample and the most recent valid calibration, the GC-MS system was inspected and corrected. Chromatographic data processing was performed manually by an experienced analyst using Agilent Chemstation Software (Agilent Technologies, Santa Clara, CA, USA). The National Institute of Standards and Technology (NIST) Library was used as the reference library. To increase the validity of measurement, we retained only those compounds with library match scores higher than 90%. Mass spectrum peaks for which proper identification was not possible because of poor library matches and a lack of confirmed retention times were then discarded. We performed standard blank correction to prevent contamination from ambient air; the concentrations of VOCs of ambient air were subtracted from the concentrations of VOCs of breath air for analysis [22, 23].

## Statistics

First, we used canonical discriminant analysis (CDA) and a forward stepwise method to build the discriminant model among (1) dust-exposed stone workers with pneumoconiosis, (2) dust-exposed stone workers without pneumoconiosis, and (3) non-dust-exposed stone workers who were primarily responsible for sales or administrative work and who did not have pneumoconiosis. Using the ILO/ICRP criteria combined with an abnormal pulmonary function test as the golden standard for pneumoconiosis, we calculated the discrimination accuracy of the breath test. We excluded the VOCs of common automobile exhaust and smoking, and we selected VOCs that were higher in the persons with pneumoconiosis who had lung crackles. A dimension reduction method was used to generate the prediction models [24]. By principal component analysis (PCA), we extracted latent components with eigenvalues >1 and obtained their factor scores. Linear combinations of these factor scores were used to generate a prediction score [25]. We generated a receiver operator characteristic (ROC) curve and calculated the area under the ROC curve (AUC) to estimate the screening accuracy of the breath test. The accuracy was validated by a 5-fold cross-validation method to obtain a mean value of model accuracy. The data were randomly divided into 5 groups. For each test, one group was removed from the set and was used as the test set, and the remaining four groups formed the training set. The model was built on the training set and was validated on the test set. We then obtained a mean value of model accuracy [26]. Statistical calculations were performed using the pROC package in R software [27] and PASW Statistics 16 (The IBM Corporation, Somers, NY, USA).

## Results

**Fig. 1:**
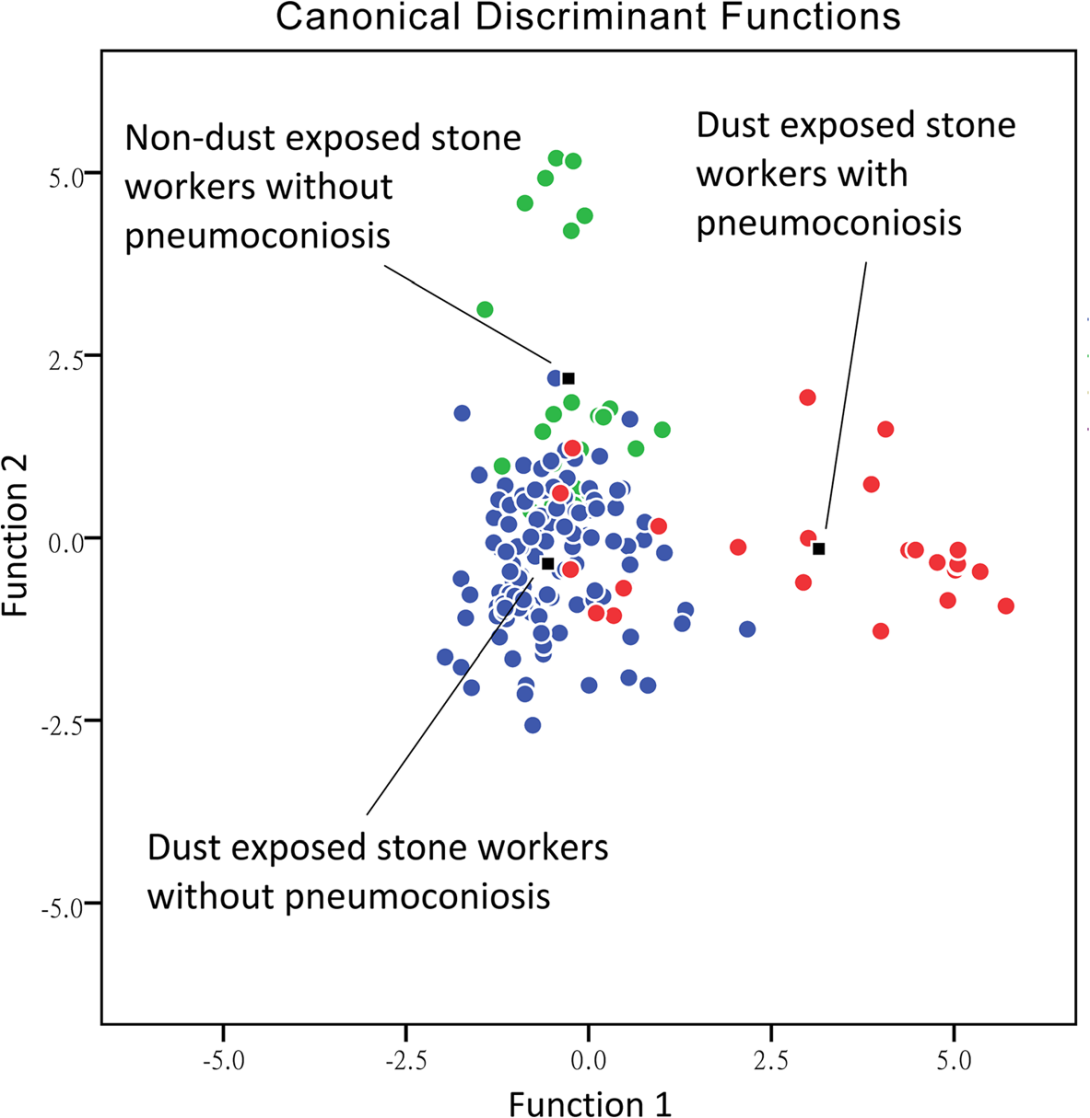
Classification of subjects by the exposure status and presence of pneumoconiosis. Legend: The subjects were classified into three groups. By canonical discriminant analysis and a forward stepwise method, 88.8% of the original grouped cases were correctly classified

**Fig. 2:**
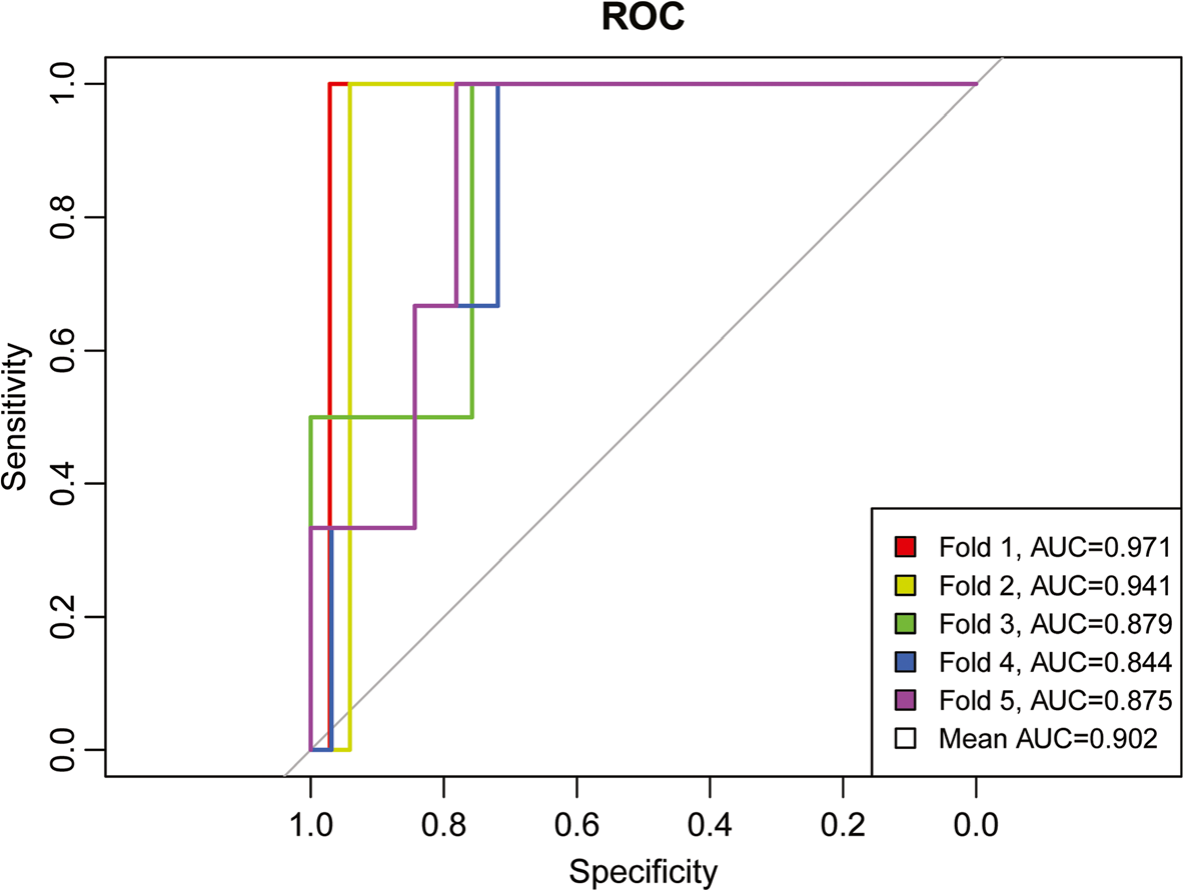
Receiver operating characteristic curves for pneumoconiosis using 5-fold cross-validation. Legend: The data were randomly divided into 5 groups. For each test, one group was removed from the set and was considered the test set, and the remaining four groups formed the training set. The model was built on the training set and was validated on the test set. The mean area under the curve (AUC) of the model is greater than 0.9, which suggests high diagnostic accuracy of the breath test

A total of 200 subjects were enrolled in the study. After excluding five subjects with a medical history of asthma with FeNO greater than 50 ppb on the examination day and 16 subjects using steroids or taking NSAIDs on the examination day, a total of 179 subjects were included in the final analysis, which included 25 cases and 154 controls. Cases had a higher proportion of males and were older than controls. The proportions of possible exposure to asbestos were higher in cases than controls (Table 1). In discriminant analysis, 88.8% of the subjects were correctly classified by their exposure and presence of pneumoconiosis (Fig. 1). We identified 195 VOCs by GC-MS. After excluding the VOCs of automobile exhaust and cigarette smoking, the PCA analysis showed that pentane, methylated heptane, and methylated cyclohexane were the major constituents identified in cases of pneumoconiosis who had lung crackle. We generated a predictive score using three factor scores. Using the Youden index to determine the best cut-off point with the largest value of (sensitivity + specificity −1) [28], the sensitivity was 100.0%, the specificity was 74.7%, the positive predicted value was 19.2%, and the negative predicted value was 100.0%*.* The predictive score had good diagnostic accuracy (AUC = 0.88, 95% CI = 0.80—0.95). The mean AUC of 5-fold cross-validation was 0.90 (Fig. 2). The concentrations of the VOCs used in the prediction model are summarized in Table 2. A significantly higher concentration of pentane was observed in cases of pneumoconiosis than in healthy controls.

**Table 1:** Characteristics of study subjects

	Cases (*n* = 25)	Controls (*n* = 154)
Men, %	68.0	46.1
Age (yrs.), mean (SD)	60.0 (9.2)	50.3 (11.8)
Duration of stone work (yrs.), mean (SD)	19.8 (14.5)	17.6 (13.8)
BMI (SD)	25.7 (3.6)	25.1 (3.9)
FVC (% of predicted) (SD)	74.3 (9.5)	93.8 (14.3)
FEV1 (% of predicted) (SD)	75.6 (9.4)	95.4 (14.4)
FEV1/FVC (%) (SD)	83.2 (6.8)	84.0 (7.7)
FEF25–75 (% of predicted) (SD)	72.9 (20.7)	87.9 (25.0)
Cigarette smoking		
Pack years, mean (SD)	39.2 (35.6)	39.0 (24.8)
Never smoked, %^a^	44.0	66.2
Former smoker, %	16.0	11.0
Current smoker, %	40.0	22.7
Passive smoking, %^b^	60.0	40.3
Possible exposure to asbestos, %^c^	96.0	73.4

^a^“Never smoked” means having smoked fewer than 20 packs of cigarettes in a lifetime or less than one cigarette per day for 1 year

^b^“Passive smoking” means having been exposed to others smoking more than three times per week for more than 6 months

^c^Occupational exposure to asbestos at ship demolition, asbestos cement, or carving asbestos-contaminated ores including nephrite, serpentine, and talc

**Table 2:** Concentrations of volatile organic compounds in breath samples

Compound	Case group		Control group		*P*-value^a^
	Mean	Std. Error of Mean	Mean	Std. Error of Mean	
Methyl chloride	12.0	5.2	5.4	1.0	0.02
Acetone	722.0	106.8	526.9	43.6	0.01
Pentane	2.2	0.8	1.2	0.4	0.03
Hexane	0.3	0.1	0.5	0.2	0.04
Butanal, 3-methyl-	0.5	0.4	0.2	0.1	0.01
1,4-Cyclohexadiene, 1-methyl-4-(1-methyl)	0.4	0.3	0.2	0.2	0.02
Phenylethyl alcohol	13.5	13.5	0.03	0.03	<0.01
Cyclohexane, 1-methylene-3-(1-methylethyl)	1.2	1.1	0.4	0.3	<0.01
Bicyclo[4.1.0]heptane, 3,7,7-trimethyl-	41.0	27.2	9.7	4.7	<0.01

^a^One-tailed *P*-value of Wilcoxon signed ranks test

## Discussion

The analysis of VOCs in breath is a novel screening method in respiratory research. Pentane, C5-C7 alkanes, and methylated alkanes constitute a distinct fingerprint in the breath of pneumoconiosis patients. This study showed that exhaled breath might be used in the screening of pneumoconiosis.

This study paid particular attention to methodological issues. We used standard blank correction to eliminate contamination from ambient air. Moreover, we excluded subjects with co-morbidities or the use of medications that might have influenced exhaled VOCs. The composition of body adipose tissues may affect the measurement of lipid metabolite profiles [29]. In this study, the subjects rested for 1 h before sampling to prevent interference from exercise-induced lipid metabolites [30]. Gas-uptake pharmacokinetic studies in rats have shown the elimination half-life of pentane to be approximately 0.13 h [31]. Considering the bioaccumulation of environmental VOCs, all of our study subjects sat for at least 1 h before breath samples were collected, which allowed exogenous VOCs to be eliminated from breath air.

The significance of this study is its translation of basic breathomics research into clinical application. Breathomics is a developing area of personalized medicine based on the capture, identification, and quantification of VOC patterns in human breath and the utilization of these data as tools in the diagnosis of diseases [32]. The metabolome reflects the interaction between the genome and the environment of an organism and reveals the ultimate response of an organism to genetic alterations, disease, and environmental influences [33]. Of particular interest to metabolomics researchers are small, low-molecular-weight compounds that serve as substrates and products in various metabolic pathways [34]. In particular, the concentrations of C4-C20 alkanes in the atmosphere and in breath are very low [35]. There is a critical gap between the qualitative and quantitative analytical methods for measuring these small-molecule metabolites. In this study, the analytical methods used were in accordance with the standard U.S. Environmental Protection Agency Method TO-15 for the analysis of trace VOCs in environmental air. We used GC-MS coupled with an air pre-concentrator capable of measuring trace VOCs at the sub-ppb level and performed high-throughput measurements to extend the compound ranges from C5-C12. Because metabolites are fast-moving targets, sample preparation is the most time-consuming and important step in this process. This study used an Entech Bottle-Vac instead of a Tedlar bag for sampling because the internal surface of the Entech Bottle-Vac is similar to that of a GC column, which allows the collected VOCs to remain stable for weeks prior to analysis [36].

The oxidation of fatty acids, also known as lipid peroxidation, is an important process that results from oxidative stress [37]. Lipid bilayers are basic components of physiological cell membranes. Pentane is a metabolite of lipid peroxidation [38]. In vitro studies have shown that the straight-chain aliphatic hydrocarbons of ethane and pentane are generated from the peroxidation of polyunsaturated fatty acids in the lipid bilayers of the cell membrane when cell cultures are exposed to ROS [39]. Methylated hydrocarbons, such as 3-methyltridecane, 3-methylundecane, and 5-methylnonane, have been used as markers of lipid peroxidation [40]. In this study, C5-C7 alkanes and methylated alkanes constituted the distinctive pattern of VOCs in the breath of pneumoconiosis. The alkanes and methylated alkanes have been used as indicators of lipid peroxidation in some diseases, such as interstitial lung diseases [41], cystic fibrosis [42], chronic obstructive pulmonary disease [15], inflammatory bowel disease [43], and scleroderma [44].

Several limitations of our study should be acknowledged. Cigarette smoking might influence exhaled breath [45]. In this study, we did not exclude current smokers from the final analyses because a high proportion of the workers had a history of smoking and their exclusion would decrease the statistical power. A small sample size and significant differences in terms of age and sex between cases and controls might decrease the validity of this study. This study was based on a case-control study in a medical centre of Hualien. To apply the test to different hospitals, we feel that a multi-centre study is warranted. External validation may validate the robustness of the prediction accuracy with further health-related applications [46]. Because of the limited number of study subjects, this study applied an internal validation method and did not use an independent validation cohort for external validation. The results of this study should be interpreted conservatively. Because this study collected air from the mouth, the exhaled breath might contain both air released from the alveolar-capillary membrane and air in the dead space. The air in the respiratory dead space of the respiratory tract [47], oral cavity, or gastrointestinal tract may influence the VOCs in exhaled air [20]. In this study, all subjects refrained from eating for at least 8 h to eliminate contamination of the upper airway and oral cavity. To prevent contamination from ambient air, we used a blank correction method to correct the background concentration of VOCs in ambient air and excluded the VOCs of common automobile exhaust and smoking to decrease the influence from environmental contamination in data analyses. Future studies may adopt an inspiratory VOC filter and a mainstream carbon dioxide monitor to collect the alveolar air [48]. The selection of VOCs might be subjective across different studies, and we suggest the procedures must be standardized to ensure reproducibility.

## Conclusion

The metabolites from pathological changes in the alveolar-capillary membrane are directly released into the alveolar space and can be detected in the breath. Lipid peroxidation plays an important role in the pathogenesis of pneumoconiosis. Pentane, C5-C7 alkanes, and methylated alkanes constitute a distinct fingerprint in the breath of persons with pneumoconiosis. VOC analysis in exhaled breath could be used to screen for pneumoconiosis. A multi-centre study is warranted, and the procedures must be standardized before clinical application.

## Abbreviations

AUC: Area under curve
CDA: Canonical discriminant analysis
CVA: Cross-validated accuracy
FEF25-75: Forced expiratory flow between 25 and 75% of FVC
FeNO: Fractional exhaled nitric oxide
FEV1: Forced expiratory volume in 1 second
FVC: Forced vital capacity
GC: Gas chromatograph
MS: Mass spectrometer
PCA: Principal component analysis
PFT: Pulmonary function test
ROC: Receiver operator characteristic
ROS: Reactive oxygen species
VOCs: Volatile organic compounds
